# Fate of the Patent False Lumen of the Descending Aorta After Surgical Treatment for Acute Type 1 Aortic Dissection

**DOI:** 10.21470/1678-9741-2022-0257

**Published:** 2023-10-02

**Authors:** Emre Yaşar, Zihni Mert Duman, Barış Timur, Muhammed Bayram, Mustafa Can Kaplan, Ersin Kadiroğulları

**Affiliations:** 1 Department of Cardiovascular Surgery, Istanbul Mehmet Akif Ersoy Thoracic and Cardiovascular Surgery Training and Research Hospital, Istanbul, Turkey; 2 Department of Cardiovascular Surgery, Cizre State Hospital, Şırnak, Turkey; 3 Department of Cardiovascular Surgery, Istanbul Dr. Siyami Ersek Thoracic and Cardiovascular Surgery Training and Research Hospital, Istanbul, Turkey

**Keywords:** Thoracic Aorta, Diaphragm, Aortic Dissection, Endovascular Procedures, Treatment Outcome

## Abstract

**Introduction:**

This study aimed to investigate the factors affecting false lumen patency in the descending thoracic aorta among patients who underwent surgery for acute type 1 aortic dissection.

**Methods:**

A total of 112 patients with acute type 1 aortic dissection, with the flap below the diaphragm level, underwent surgery between January 2010 and September 2019. Of these, 60 patients who were followed up for ≥ 12 months and whose computed tomography scans were available were included in this study. The patients were divided into two groups: group I, consists of patent false lumen (n=36), and group II, consists of thrombosed false lumen (n=24). Demographic data, operative techniques, postoperative descending aortic diameters, reintervention, and late mortality were compared between the two groups.

**Results:**

The mean follow-up period of all patients was 37.6±26.1 months (range: 12-104). The diameter increase in the proximal and distal descending aorta was significantly higher in the patent false lumen group (5.3±3.7 mm vs. 3.25±2.34 mm; P=0.015; 3.1±2.52 mm vs. 1.9±1.55 mm; P=0.038, respectively). No significant difference in terms of hypertension was found between the two groups during the follow-up period (21 patients, 58.3% vs. 8 patients, 33.3%; P=0.058). A total of 29 patients (48.3%) were found to be hypertensive in the postoperative period.

**Conclusion:**

After surgical treatment for acute type 1 aortic dissection, patients should be monitored closely, regardless of whether the false lumen is patent or thrombosed. Mortality and reintervention can be seen in patients with patent false lumen during follow-up.

## INTRODUCTION

**Table:** 

Abbreviations, Acronyms & Symbols	
AXA	= Axillary artery
COPD	= Chronic obstructive pulmonary disease
CPB	= Cardiopulmonary bypass
CT	= Computed tomography
FA	= Femoral artery
HD	= Hemodynamically
LVEF	= Left ventricular ejection fraction
PFL	= Patent false lumen
TEVAR	= Thoracic endovascular aortic repair

Despite significant advances in surgical techniques and technology, acute type 1 aortic dissection remains a cardiovascular condition with a high risk for mortality. The early postoperative mortality rate lies between 7% and 30%^[1-3]^. Emergency surgical treatment is only the initial step for these patients, who might develop dilatation of the residual distal aorta with an imminent risk of aortic rupture^[4]^. The analysis of preoperative and postoperative computed tomography (CT) images helps in determining an optimal treatment strategy to prevent aortic dilatation and facilitate to make timely decision for surgical or endovascular interventions[5]. Patent false lumen (PFL) is one of the significant risk factors for secondary descending aortic enlargement^[6]^. The PFL rate is reported to lie between 50% and 62%^[1,5]^. Studies have reported a higher incidence of aortic rupture and reoperation and lower survival depending on the PFL^[7]^. Procedures such as systematic extended total arch replacement or elephant trunk were recommended to decrease the incidence of PFL^[8-10]^. However, the causes and possible outcomes of residual PFL are still not fully understood. In the presence of a thrombosed false lumen, the enlargement of the descending aorta and the need for reoperation suggests that PFL is not the only factor for reintervention.

In this study, we compared CT images obtained during the follow-up of patients with acute type 1 aortic dissection, whose dissection flap extended under the diaphragm. We compared the reoperation rates, late mortality, and enlargement of the descending aortic diameter among patients with patent and thrombosed lumen.

## METHODS

**Fig. 1:**
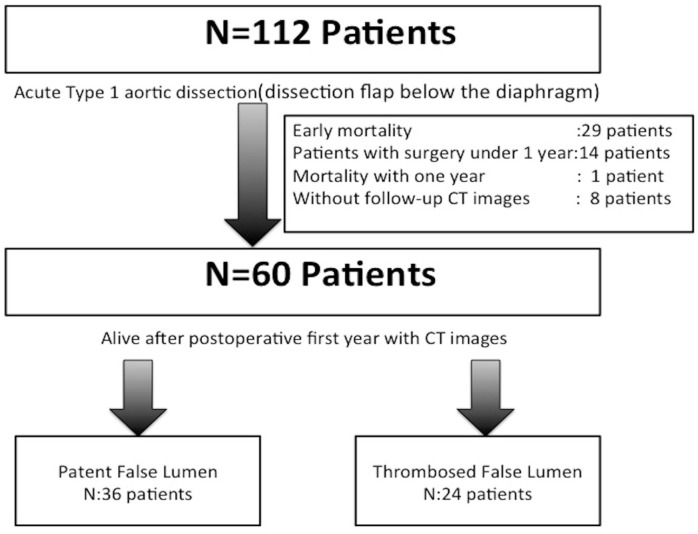
Exclusion criteria and case distribution. CT=computed tomography.

**Fig. 2:**
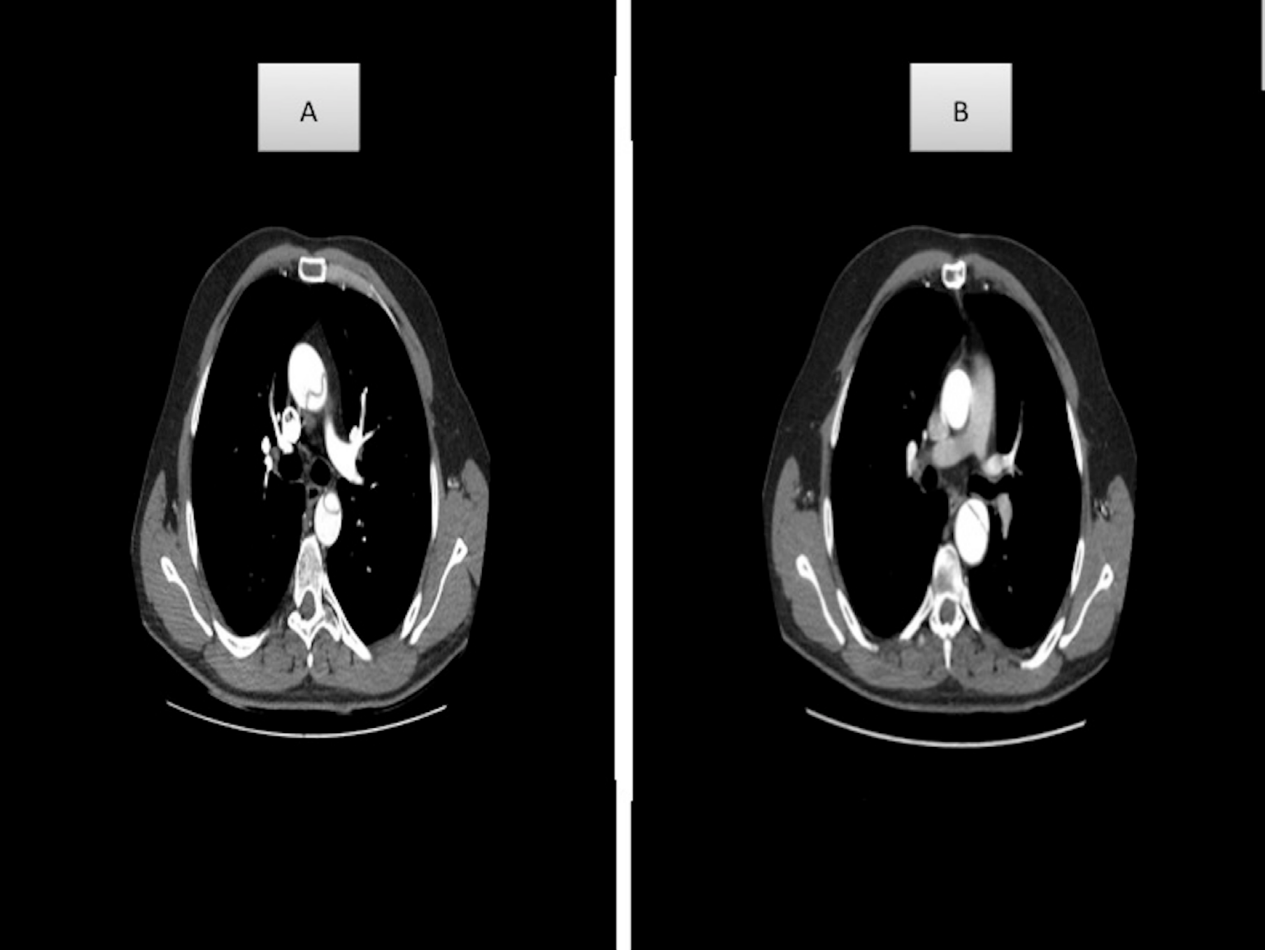
(A) Preoperative thoracic aorta, patent false lumen; (B) postoperative thoracic aorta, patent false lumen.

**Fig. 3:**
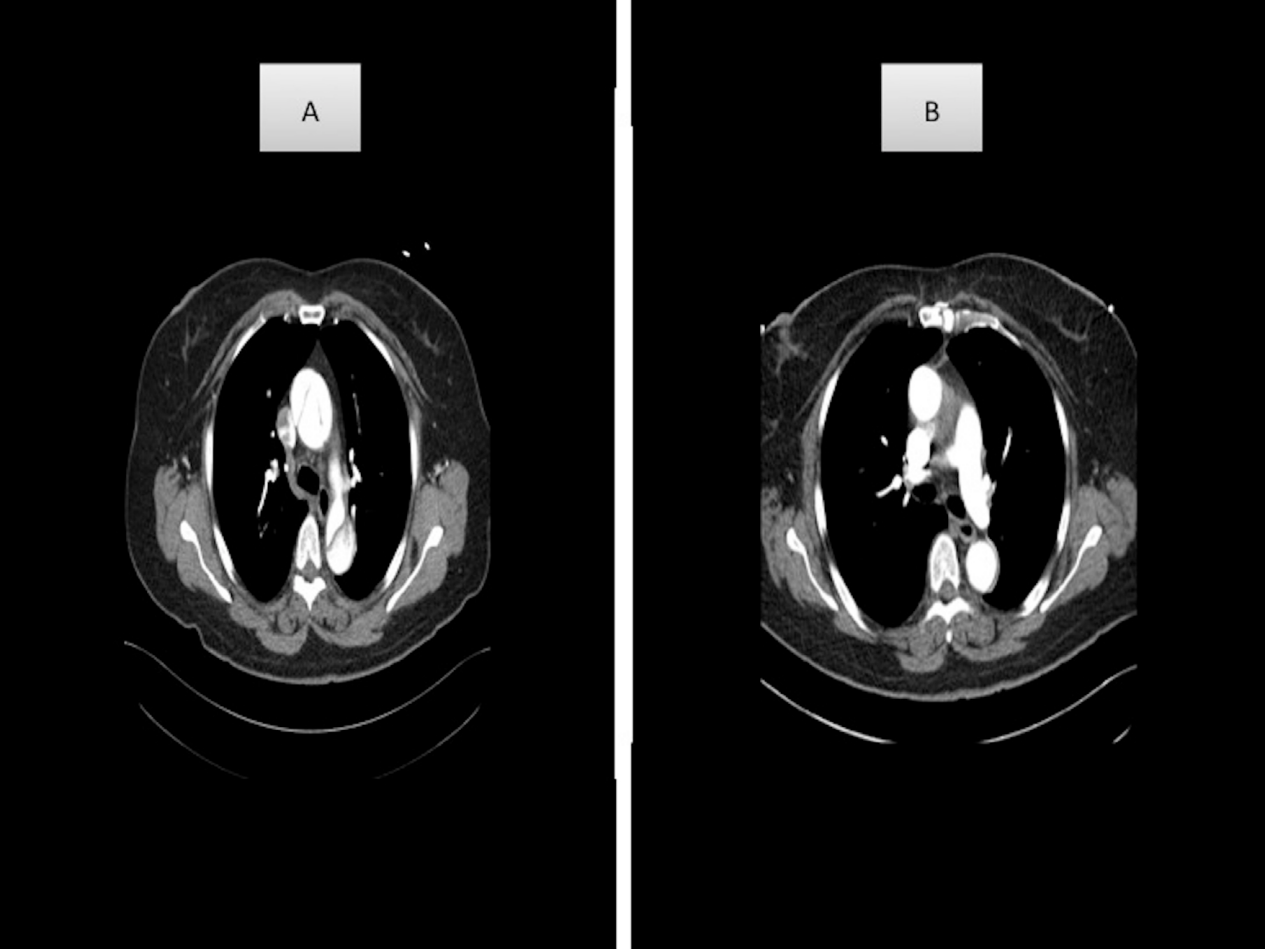
(A) Preoperative thoracic aorta, patent false lumen; (B) postoperative thoracic aorta, thrombosed false lumen.

A total of 112 consecutive patients underwent emergency aortic surgery for acute type 1 aortic dissection, with dissection flap below the diaphragm level, at our hospital between January 2010 and September 2019. Patients who were alive and who had CT images at least 12 months postoperatively were included in the study. Patients with early mortality, follow-up period < 12 months, patients who died within one year, and patients who could not have CT images during follow-up were excluded from the study (Figure 1). A total of 60 survivors had follow-up CT scan images available in the postoperative 12 months. PFL or partial thrombosed false lumens revealed by CT angiography were considered as PFL (Figure 2). If the false lumen was completely thrombosed, it was considered as a thrombosed false lumen (Figure 3). These patients were divided into two groups according to PFL (group I, n=36) and thrombosed false lumen (group II, n=24). In each group, we analyzed the demographic, intraoperative, and postoperative data, late mortality, reoperation rates, and the diameter of the proximal and distal descending thoracic aorta. Patients who were in the pre-shock state (systolic blood pressure < 90 mmHg or receiving inotrope) were considered hemodynamically unstable. Patients with a creatinine level > 1.8 mg/dL in the postoperative period were considered to have acute renal failure. Early mortality was considered as mortality within the first 30 days. The patients’ data were accessed through hospital records and via direct communication with the patients. This retrospective study protocol was approved by the local ethics committee. The study was conducted in accordance with the principles of the Declaration of Helsinki.

### Surgical Procedure

All patients underwent ascending aorta surgery within 24 hours of symptom onset. The algorithm of the guidelines was used to make the decision to perform surgery^[11]^. All procedures were performed under general anesthesia with single-lumen intubation. The surgical procedure consisted of median sternotomy with standard cardiopulmonary bypass (CPB) via axillary artery (AXA) or femoral artery (FA) cannulation or both. The right atrium was cannulated with a double-stage cannula for venous drainage, and the left ventricle was vented through the right upper pulmonary vein. Arterial cannulation was usually performed through the FA in the first year of our clinic. After CPB was established, systemic cooling was started immediately. In the last five years, AXA has been preferentially used as the route for systemic perfusion, except in patients who presented with shock (systolic blood pressure < 70 mmHg) and required immediate CPB via FA cannulation. In some patients, we used both AXA and FA methods. Before the antegrade cerebral perfusion, vascular clamps were placed on the brachiocephalic trunk, left common carotid artery, and left subclavian artery, and cerebral perfusion with oxygenated blood was initiated via AXA at a flow rate of 10 mL/kg/min and temperature of 26°C. After aortotomy, a distal anastomosis was performed in the circulatory arrest period. Entry tear was removed completely in all patients. After termination of the distal anastomosis, the graft was clamped following deairing, and systemic heating was initiated in all cases.

In both techniques, a proximal anastomosis was performed, while systemic heating was applied to the anastomosed graft after the cross-clamp. Surgical procedures consisted of hemiarch replacement with replacement of the ascending aorta with or without aortic root surgery (Bentall operation). Aortic valve resuspension and coronary artery bypass grafting were performed in patients when indicated. Total aortic arch replacement was reserved for patients who had extensive aneurysmal involvement of the arch or proximal descending aorta, and for patients with type 1 aortic dissection with an entry site or reentry tear in the aortic arch.

### Follow-up

Patients who were operated for acute type 1 aortic dissection without mortality for at least 12 months postoperatively and had CT images available were included in the study. Patients with late mortality and reintervention after one year were divided into two groups according to the state of false lumen in the CT images. The measurements in the descending aorta were obtained by measuring the aortic diameters at the level of the pulmonary bifurcation for the proximal section, and at the level of the diaphragm for the distal sections. Dilatation measurements were made by taking the difference between the preoperative CT image and the last CT image taken. While taking the postoperative CT images, patients with hypertension who were not followed up regularly were considered as being hypertensive.

### Statistical Analysis

Statistical analyses of the data were performed using the IBM Corp. Released 2012, IBM SPSS Statistics for Windows, version 22.0, Armonk, NY: IBM Corp. software package. Continuous variables are expressed as mean ± standard deviation, and categorical variables are presented as number and percentages. Kolmogorov-Smirnov test was used to test normal distribution of categorical variables. Comparisons of continuous and categorical variables between the groups were made using Student’s unpaired *t*-test or Mann-Whitney U test, and the Chi-square (χ^^2^^) test, respectively. Univariate and multivariate analyses were used to determine factors significantly related to PFL. A two-tailed *P*-value < 0.05 was considered statistically significant. The data were prospectively collected in a computerized database.

## RESULTS

Demographical and baseline characteristics are summarized in Table 1. The two groups were similar in terms of baseline and demographical characteristics. No significant difference was found in terms of the number of patients whose hemodynamics were stable during surgery (25 patients, 69.4% *vs.* 17 patients, 70.8%; *P*=0.908).

**Table 1:** Patients’ demographic characteristics.

Parameters	Patent false lumen (n=36)	%	Thrombosed false lumen (n=24)	%	*P*-value
Age (years)	53±9.7		49.5±10.3		0.202
Male sex	30	83.3	21	87.5	0.658
Hypertension	30	83.3	19	79.2	0.683
Diabetes	5	13.9	7	29.2	0.147
Renal failure	4	11.1	2	8.3	0.725
COPD	11	30.6	6	25	0.640
Coronary artery disease	7	19.4	6	25	0.609
Marfan syndrome	1	2.8	1	4.2	0.769
LVEF (%)	57.7±4.1 (45-65)		55.4±4.7 (40-65)		0.062
Patient transferred from another clinic	21	58.3	12	50	0.525
Preoperative state					
HD stable	25	69.4	17	70.8	0.908
HD instable	11	30.6	6	25.0	0.640
Resuscitation	0	0	1	4.2	0.217
Pericardial tamponade	6	16.7	2	8.3	0.352

COPD=chronic obstructive pulmonary disease; HD=hemodynamically; LVEF=left ventricular ejection fraction

Intraoperative data is presented in Table 2. The duration of CPB was significantly higher in the PFL group (167.9±49.6 min *vs.* 204±68.9 min; *P*=0.021). However, no significant difference was found between the two groups in terms of cross-clamping times (86.6±40.1 min *vs.* 104.9±40.8 min; *P*=0.092). There was no significant difference in terms of surgical techniques for both groups.

**Table 2:** Intraoperative outcomes.

Parameters	Patent false lumen (n=36)	%	Thrombosed false lumen (n=24)	%	*P*-value
Cardiopulmonary bypass time (min)	167 .9±49.6 (278-71)		204±68.9 (360-110)		0.021
Cross-clamping time (min)	86.6 ±40.1 (166-29)		104.9±40.8 (200-33)		0.092
Total circulatory arrest	19	52.8	15	62.5	0.457
Antegrade cerebral perfusion	17	47.2	9	37.5	0.457
Axillary artery cannulation	15	41.7	10	41.7	1.000
Femoral artery cannulation	15	41.7	12	50	0.525
Femoral-axillary canulation	6	16.7	2	8.3	0.352
Patients requiring arch replacement					
Hemiarch replacement	5	13.9	4	16.7	w0.768
Total aortic arch replacement	3	8.3	4	16.7	0.325
Ascending aortic replacement technique					
Bentall procedure	15	41.7	10	41.7	1.000
Supracoronary graft interposition	21	58.3	14	58.3	1.000
Coronary artery bypass grafting	2	5.6	0	0	0.240
Aortic valve resuspension	2	5.6	2	8.3	0.673

Postoperative complications are shown in Table 3. No significant difference was found between the two groups in terms of postoperative complications. There was no significant difference between the two groups in terms of postoperative stroke (0 patient *vs.* one patient, 4.2%; *P*=0.217) and transient paraparesis (two patients, 5.6% *vs.* five patients, 20.8%; *P*=0.071). There was no statistically significant difference between the two groups in terms of warfarin use (17 patients, 47.2% *vs.* 11 patients, 45.8%; *P*=0.916).

**Table 3:** Postoperative outcomes.

Parameters	Patent false lumen (n=36)	%	Thrombosed false lumen (n=24)	%	*P*-value
Duration of intensive care unit stay (day)	7.7±5.2 (1-45)		11.4±8 (1-56)		0.337
Duration of hospital stay (day)	11.6±10.9 (5-60)		16.7±17 (5-85)		0.170
Pulmonary complications	3	8.3	6	25	0.077
Arrhythmia					
Atrial fibrillation	3	8.3	1	4.2	0.526
Heart block	3	8.3	1	4.2	0.526
Re-exploration					
Bleeding	10	27.8	7	29.2	0.907
Tamponade	2	5.6	2	8.3	0.673
New-onset neurologic dysfunction					
Permanent	0	0	1	4.2	0.217
Temporary	2	5.6	5	20.8	0.071
Acute renal failure					
Elevated creatinine levels	6	16.7	2	8.3	0.352
Dialysis	2	5.6	2	8.3	0.673
Warfarin use	17	47.2	11	45.8	0.916

Table 4 summarizes follow-up of patients after discharge. No significant difference was found between the two groups in terms of follow-up times (37.5±27.6 months [range, 12-104] *vs.* 37.7±24.6 months [range, 12-102]; *P*=0.983). No difference was found between the two groups in terms of reintervention rate (four patients, 11.1% *vs.* two patients, 8.3%; *P*=0.725). Two of these four patients in the PFL group were treated with open Crawford extent II thoracoabdominal aortic aneurysm repair while the other two received treatment with thoracic endovascular aortic repair (TEVAR). TEVAR were performed in two patients who underwent reintervention in the thrombosed false lumen group. There was no significant difference between the two groups in terms of late mortality (one patient, 2.8% *vs.* two patients, 8.3%; *P*=0.333). The only patient death in the PFL group was because of rupture of the enlargement in the proximal part of the descending aorta. Two patients in the thrombosed false lumen group had long-term mortality due to respiratory failure and myocardial infarction.

**Table 4:** Follow-up.

Parameters	Patent false lumen (n=36)	%	Thrombosed false lumen (n=24)	%	*P*-value
Follow-up (months)	37.5±27.6 (12-104)		37.7±24.6 (12-102)		0.983
Reintervention	4	11.1	2	8.3	0.725
Surgical	2	5.6	0	0	0.240
Endovascular	2	5.6	2	8.3	0.673
Late mortality	1	2.8	2	8.3	0.333
Progression of aortic disease in CT					
Increase in proximal descending aortic diameter (mm)	5.3±3.7 (1-13)		3.25±2.34 (0-11)		0.015
Increase in distal descending aortic diameter (mm)	3.1±2.5 (0-9)		1.9±1.55 (0-7)		0.038
Postoperative hypertension	21	58.3	8	33.3	0.058

CT=computed tomography

Enlargement of the proximal descending aorta was significantly higher in the PFL group (5.3±3.69 mm [range, 1-13] *vs.* 3.25±2.34 mm [range, 0-11]; *P*=0.015). The amount of enlargement in the distal descending aorta was also significantly higher in the PFL group (3.1±2.5 mm [range, 0-9] *vs.* 1.9±1.55 mm [range, 0-7]; *P*=0.038). No significant difference was found between the two groups in terms of hypertension during the follow-up period (21 patients, 58.3% *vs.* eight patients, 33.3%; *P*=0.058). A total of 29 patients (48.3%) were found to be hypertensive in the postoperative period.

## DISCUSSION

Although a few studies have focused on PFL of the descending aorta, its etiopathogenesis has not been clearly defined in the literature. In the present study, we investigated the factors that may cause false lumens to be patent or thrombosed after surgical treatment of acute type 1 aortic dissection, and the conditions that may be encountered in postoperative follow-up. Although the PFL patients showed more expansion in the proximal and distal descending aorta, the effect on reintervention and late mortality has not been fully proven. In thrombosed false lumen patients, enlargement of the aorta and reintervention in the postoperative follow-up demonstrates that the pathology continues in the dissection patients after surgery.

PFL might occur even after ascending aorta surgery in patients with acute type 1 dissection^[12,13]^. The possibility of detecting postoperative PFL was reported as 50-64%^[1,5]^. Similar to the literature, the overall PFL rate in the acute type 1 dissection patient group was 60% (36/60) in our study, according to follow-up CT scans^[14,15]^. In these studies, PFLs were reported to have an influence on mortality and reintervention. Importantly, it is mandatory for patients who are diagnosed with PFL in the postoperative period to receive careful follow-up in order to prevent rupture of the proximal descending aorta. Since severe aortic dilatation results in a tendency to rupture, increase in the diameter of the descending aorta (for each 5 mm) is a predictor of mortality^[16]^. In the PFL group in this study, the amount of enlargement in the proximal and distal descending aorta was significantly higher than in the thrombosed false lumen group. However, the continued expansion of the proximal and distal aorta among patients in the thrombosed false lumen group indicates that postoperative aortic pathology continues.

The decision to perform reintervention in a stable, asymptomatic patient is another vital issue. For timely reoperation or endovascular intervention, comorbidities, aortic diameter, and the status of the residual false lumen must be taken into account. Kimura et al.^[1]^ reported that satisfactory results of reoperation in the distal aorta and no perioperative mortality occurred among eight patients who underwent reoperation. They observed a relatively low incidence of distal reoperation (89.5% freedom from reoperation at 10 years). In our patient series, a total of six patients (10%) needed reintervention (reoperation or endovascular intervention). The observation in all patients who underwent reintervention was enlargement of the proximal descending aorta. We did not observe any long-term pathology in the proximal ascending aorta and aortic root.

Zierer et al.^[17]^ reported that, in 85% of patients undergoing reintervention in the long-term follow-up of acute aortic dissection, the procedure was performed under elective conditions. In our study, all patients who underwent reintervention were operated under elective conditions, and none of the patients who underwent reintervention died. One patient in the PFL group developed late mortality before surgery due to rupture of the descending aorta. In the same study, risk factors other than PFL were identified for reintervention^[17]^. These risk factors included aortic diameter > 40 mm in the distal anastomosis line, young age, connective tissue disease, and high postoperative systolic blood pressure. In our study, no patient exceeded 40 mm in the distal anastomosis line during the surgery. There was no significant difference in patients with Marfan syndrome in both groups (one patient, 2.8% *vs.* one patient, 4.2%; *P*=0.769). The proportion of patients who stopped antihypertensive treatment or were diagnosed with postoperative hypertension despite receiving treatment was determined as 48.3% of the study group. However, there was no significant difference between the two groups in terms of postoperative systolic blood pressure elevation (58.5% *vs.* 33.3%; *P*=0.058). In the study group, six patients with reintervention were found to have high systolic blood pressure when the reintervention decision was made. In our view, the most important factor causing patients’ false lumen to remain patent is discontinuation of antihypertensive treatments or insufficient treatment in the postoperative period. This is also demonstrated by the fact that all our patients who underwent reintervention had postoperative hypertension.

Some authors recommend systematic extension or total arch replacement for the initial surgical management of acute type 1 aortic dissection, irrespective of the site of entry, to decrease the incidence of residual PFL^[1,9]^. Omura et al.^[18]^ reported that the rate of reintervention was lower in patients who underwent total aortic arch replacement without increasing early mortality. However, Leontyev et al.^[5]^ reported no significant influence of total arch replacement compared with isolated ascending aorta and partial arch replacement on PFL. Therefore, we performed total aortic arch replacement only upon detecting intimal tears during the surgical exploration but we did not perform this complex surgical technique in other conditions.

Currently, there are new endovascular technological solutions for the prevention of PFL in patients after surgery for acute type 1 aortic dissection. Esposito et al.^[19]^ treated acute aortic dissection with hybrid methods using the Lupiae technique. In this technique, patients underwent endovascular procedures in cases of malperfusion development (spinal, organ, limb) in the acute period of the postoperative follow-up, proximal descending aorta > 46 mm in diameter, presence of PFL, and false lumen of 22 mm in the chronic period. The study reported that the rate of false lumen thrombosis was 92% after this technique.

Recently, the frozen elephant trunk technique has been widely used. Gorlitzer et al.^[20]^, in their study with 14 patients, reported that the false lumen became completely thrombosed in all patients with acute and chronic dissection three months after surgery. A large international registry reported that the rate of complete false lumen thrombosis was 76% in patients who underwent surgery with frozen elephant trunk implantation, with no increase in in-hospital death rates in patients with acute type 1 aortic dissection compared with all other patients who underwent total arch replacement^[20,21]^. In another study, it was reported that the aortic dilatation of the proximal descending aorta stopped and even regressed with the elephant trunk technique^[22]^. Shrestha et al.^[23]^ used the frozen elephant trunk technique in patients with acute type 1 aortic dissection. In this study, they suggested that a surgeon should use the frozen elephant trunk technique for at least 20 cases of elective aortic surgery before using it in acute aortic dissection surgeries.

Preventza et al.^[24]^ reported that the PFL ratio was low, and long-term survival was better by surgical suturing of the endovascular graft to the descending aorta antegradely during the surgery. Moreover, in another similar study, reoperation rates were found to be low in cases where extended surgery and endovascular graft were combined^[25]^.

Some patients needed warfarin for anticoagulation due to mechanical aortic valve prosthesis or atrial fibrillation in the postoperative period. The effect of anticoagulation on false lumen patency is controversial. Öztürk et al.^[26]^ and Song et al.^[27]^ showed that anticoagulation does not increase false lumen patency. Vendramin et al.^[28]^ showed that anticoagulation influences false lumen patency, and that anticoagulation is not a risk factor for late mortality or reinterventions. In our study, anticoagulation did not show any effect on false lumen patency.

In the future, it is predicted that hybrid approaches will increase in acute type 1 aortic dissection surgeries. The purpose of surgeries will not only be to save lives, but also to prevent morbidity and mortality that may be encountered in long-term follow-up.

### Limitations

Our study has several limitations. The retrospective design of the study, its limited number of patients, and the short follow-up time are the main limitations. In addition, since 50% of the patients in our study group were transferred from neighboring provinces, a regular follow-up could not be performed in our hospital in the postoperative period. Moreover, there is a difference between the postoperative tomography times.

## CONCLUSION

Since emergency surgery for type 1 aortic dissection is a life-saving surgery, a fast and an effective procedure may be required during the surgery. However, the course of false lumens is very important during postoperative long-term follow-up. Aortic enlargement is more common in patients with PFLs. Therefore, postoperative follow-up should be done in the early periods, and blood pressure control should be regulated.

## Abbreviations

AXA: = Axillary artery
COPD: = Chronic obstructive pulmonary disease
CPB: = Cardiopulmonary bypass
CT: = Computed tomography
FA: = Femoral artery
HD: = Hemodynamically
LVEF: = Left ventricular ejection fraction
PFl: = Patent false lumen
TEVAR: = Thoracic endovascular aortic repair
